# Risk Factors and Prevalence of Dilated Cardiomyopathy in Sub-Saharan Africa: Protocol for a Systematic Review

**DOI:** 10.2196/18229

**Published:** 2021-01-21

**Authors:** Lulu Said Fundikira, Pilly Chillo, Linda W van Laake, Reuben Kato Mutagaywa, Amand Floriaan Schmidt, Appolinary Kamuhabwa, Gideon Kwesigabo, Folkert W Asselbergs

**Affiliations:** 1 Department of Cardiology Division of Heart and Lungs University Medical Centre Utrecht, Utrecht University Utrecht Netherlands; 2 Muhimbili University of Health and Allied Sciences Dar es Salaam United Republic of Tanzania; 3 Institute of Cardiovascular Science Faculty of Population Health Sciences University College London London United Kingdom; 4 Health Data Research UK and Institute of Health Informatics University College London London United Kingdom

**Keywords:** dilated cardiomyopathy, cardiomyopathy, heart failure, cardiovascular risk factors, sub-Saharan Africa

## Abstract

**Background:**

Cardiomyopathies, defined as diseases involving mainly the heart muscles, are linked to an estimated 5.9 of 100,000 deaths globally. In sub-Saharan Africa, cardiomyopathies constitute 21.4% of heart failure cases, with dilated cardiomyopathy (DCM) being the most common form. The etiology of DCM is heterogeneous and is broadly categorized as genetic or nongenetic, as well as a mixed disease in which genetics interact with intrinsic and environmental factors. Factors such as age, gender, family history, and ethnicity are nonmodifiable, whereas modifiable risk factors include poor nutrition, physical inactivity, and excessive alcohol consumption, among others. However, the relative contribution of the different risk factors to the etiology of DCM is not known in sub-Saharan Africa, and the prevalence of DCM among heart failure patients has not been systematically studied in the region.

**Objective:**

The aim of this review is to synthesize available literature from sub-Saharan Africa on the prevalence of DCM among patients with heart failure, as well as the literature on factors associated with DCM. This paper outlines the protocol that will be followed to conduct the systematic review.

**Methods:**

A limited search of the PubMed database will be performed to identify relevant keywords contained in the title, abstract, and subject descriptors using initial search terms “heart failure,” “cardiomyopathy,” and “sub-Saharan Africa.” These search terms and their synonyms will then be used in an extensive search in PubMed, and will address the first research question on prevalence. To address the second research question on risk factors, the terms “heart failure,” “cardiomyopathy,” and “cardiovascular risk factors” in “Sub-Saharan Africa” will be used, listing them one by one. Articles published from 2000 and in the English language will be included. Indexed articles in PubMed and Embase will be included, as well as the first 300 articles retrieved from a Google Scholar search. Collected data will be organized in Endnote and then uploaded to the Rayyan web app for systematic reviews. Two reviewers will independently select articles against the inclusion criteria. Discrepancies in reviewer selections will be resolved by an arbitrator. PRISMA (Preferred Reporting Items for Systematic Reviews and Meta-Analyses) guidelines for reporting systematic reviews will be applied. A map of sub-Saharan Africa with colors to show disease prevalence in each country will be included. For quantitative data, where possible, odds ratios (for categorical outcome data) or standardized mean differences (for continuous data) and their 95% CIs will be calculated.

**Results:**

The primary outcomes will be the prevalence of DCM among patients with heart failure and cardiovascular risk factors associated with DCM in sub-Saharan Africa. The literature search will begin on January 1, 2021, and data analysis is expected to be completed by April 30, 2021.

**Conclusions:**

This review will provide information on the current status of the prevalence and associated factors of DCM, and possibly identify gaps, including paucity of data or conflicting results that need to be addressed to improve our understanding of DCM in sub-Saharan Africa.

**International Registered Report Identifier (IRRID):**

PRR1-10.2196/18229

## Introduction

### Background

An interesting phenomenon is unfolding in sub-Saharan Africa due to globalization and urbanization. The region traditionally plagued with infectious diseases is currently facing a double burden of disease as evidenced by the rise of noncommunicable diseases, mainly cardiovascular diseases (CVDs) [1]. The increase in CVD incidence has resulted in a growing burden of heart failure in sub-Saharan Africa [2], a trend that is expected to increase over time [3].

Cardiomyopathies, which are diseases affecting mainly the heart muscles, are a common cause of heart failure worldwide, and represent a significant cause of morbidity and mortality. In 2010, cardiomyopathies were estimated to cause mortality in up to 5.9 of 100,000 individuals globally and most likely are underdiagnosed [4,5]. In sub-Saharan Africa, a contemporaneous systematic review and meta-analysis of the etiology of heart failure performed by Agbor et al [6] showed that cardiomyopathies (all forms) constituted 21.4% (18.2%-40.2%) of all heart failure cases, second only to hypertensive heart disease as a cause of heart failure. Among the different types of cardiomyopathies, dilated cardiomyopathy (DCM) is by far the most common in sub-Saharan Africa [7-9].

DCM is defined as the presence of left or biventricular dilatation and contractile dysfunction in the absence of abnormal loading conditions (such as hypertension or valve disease) or coronary artery disease that is sufficient to cause global contractile impairment [10]. The etiology of DCM is diverse and heterogeneous, including genetic mutations, infections, and autoimmunity, although in most instances the etiology cannot be completely identified [5]. The European Society of Cardiology (ESC) classifies DCM as familial or nonfamilial, in which familial cases usually have a genetic cause [11]. However, the American Heart Association classifies DCM as genetic, acquired, or mixed [12]. A revised definition of DCM by the ESC Working Group on Myocardial and Pericardial Diseases highlighted the heterogeneous nature of the disease that can broadly be grouped as genetic or nongenetic, although there are some circumstances in which a genetic predisposition interacts with intrinsic or environmental factors to form a clinical picture seen in DCM [13]. The presence of nonmodifiable cardiovascular risk factors such as family history, age, ethnicity, and gender, as well as modifiable risk factors such as hypertension, diabetes, tobacco use, physical inactivity, poor nutrition, excessive alcohol consumption, high cholesterol, and obesity increase the probability of developing CVD and heart failure [14,15].

However, the relative contribution of the different risk factors to the etiology of DCM is not known in sub-Saharan Africa, and the prevalence of DCM among heart failure patients has not been systematically studied in the region.

With the increasing recognition of DCM as a heterogeneous and diverse disease [11,12,16], it is important to understand the contribution of the different cardiovascular risk factors to the clinical presentation of DCM. Identifying risk factors associated with DCM may bring about insightful management consequences, including medical counseling directed to patients and their relatives to avoid or manage the modifiable risk factors so as to halt or prolong the course of DCM. This review will systematically study the available data published from 2000 onward to capture the current situation in sub-Saharan Africa with regard to the risk factors and prevalence of DCM in patients with heart failure. This time period has been selected to reflect the current definitions of DCM [11-13].

### Aim of This Review

The aim of this review is to determine the prevalence of DCM and its associated risk factors among patients with heart failure in sub-Saharan Africa.

### Review Questions

The specific review questions to be addressed are: (1) What is the prevalence of DCM in patients with heart failure in sub-Saharan Africa? (2) What are the associated risk factors for DCM in patients with heart failure in sub-Saharan Africa?

## Methods

### Inclusion Criteria

All full-text articles from observational studies (cross-sectional, cohort, retrospective, or prospective) that meet the search criteria, published in the English language from January 1, 2000 to December 31***,*** 2020 will be included in this review.

### Exclusion Criteria

This review will exclude case reports, editorials, comments or expert opinions, as well as letters of study subjects due to lack of peer review. In addition, articles published in a language other than English will be excluded. Qualitative studies will also be excluded.

### Search Strategy

A limited search of PubMed will be performed to identify relevant keywords contained in the title, abstract, and subject descriptors. The initial search terms will be “heart failure,” “cardiomyopathy,” and “sub-Saharan Africa”; these search terms and their synonyms will then be used in an extensive search in PubMed. This search will be applied to answer question 1 on prevalence. Thereafter, a search will be performed to answer question 2 using the terms “heart failure,” “cardiomyopathy,” and the risk factors of interest, which are age, gender, ethnicity, family history, hypertension, diabetes, tobacco use, physical inactivity, poor nutrition, excessive alcohol consumption, high cholesterol, and obesity, in “sub-Saharan Africa.” Filters will be added to narrow down to articles published from 2000 and in the English language. Indexed articles in PubMed and Embase will be included. Taking into account that some journals in Africa may not be indexed in PubMed, searches in Google Scholar will also be performed, and the first 300 articles will be included. The detailed search terms following the PICO (Patient/Population/Problem, Intervention/Prognostic Factor, Comparison, Outcome) format are as follows:

P: “heart failure” [MeSH Terms] OR (“heart” [All Fields] AND “failure” [All Fields]) OR “heart failure” [All Fields] in “Sub-Saharan Africa” OR “Africa” OR “Saharan” OR ((Angola OR Burundi OR DRC OR Cameroon OR Central Africa Republic OR Chad OR Republic of Congo OR Equatorial Guinea OR Gabon OR Kenya OR Nigeria OR Rwanda OR Sao Tome and Principe OR Tanzania OR Uganda OR South Sudan OR Eritrea OR Ethiopia OR Botswana OR Comoro OR Lesotho OR Madagascar OR Malawi OR Mauritius OR Mozambique OR Namibia OR Seychelles OR South Africa OR Swaziland OR Zambia OR Zimbabwe OR Benin OR Mali OR Burkina Faso OR Cape Verde OR Ivory Coast OR Gambia OR Ghana OR Guinea OR Guinea Bissau OR Liberia OR Niger OR Mauritania OR Senegal OR Sierra Leone OR Togo))).

I: (“cardiovascular system” [MeSH Terms] OR (“cardiovascular” [All Fields] AND “system” [All Fields]) OR “cardiovascular system” [All Fields] AND (“risk factors” [MeSH Terms] OR (“risk” [All Fields] AND “factors” [All Fields]) OR “risk factors” [All Fields]).

C: “age” OR “gender” OR “family history” OR “hypertension” OR “diabetes” OR “tobacco use” OR “physical inactivity” OR “poor nutrition” OR “excessive alcohol consumption” OR “high cholesterol” OR “obesity”.

O: “cardiomyopathy, dilated” [MeSH Terms] OR (“cardiomyopathy” [All Fields] AND “dilated” [All Fields]) OR “dilated cardiomyopathy” [All Fields] OR (“dilated” [All Fields] AND “cardiomyopathy” [All Fields]).

### Risk of Bias and Study Quality

Identified studies that meet the inclusion criteria will be assessed independently for methodological validity by two reviewers prior to inclusion in the final analysis using the Risk Of Bias In Non-randomized Studies of Interventions (ROBINS-I) assessment tool, version for cohort-type studies (see Multimedia Appendix 1) [17].

### Data Collection

Full copies of articles identified by the search, and considered to meet the inclusion criteria based on their title, abstract, and subject descriptors will be obtained for data synthesis. The collected data will be organized in Endnote reference manager and subsequently uploaded to the Rayyan web app for systematic reviews to allow for adequate sorting [18]. Two reviewers will independently select articles against the inclusion criteria. Discrepancies in reviewer selections will be resolved by a third author (arbitrator) prior to the selected articles being retrieved. A data extraction tool will be developed specifically for quantitative research data extraction based on the work of the Cochrane Collaboration and the Centre for Reviews and Dissemination, as shown in Multimedia Appendix 2. Two reviewers will independently perform data extraction. In cases of missing data, the corresponding authors of the study will be approached once by email.

### Data Synthesis

The Preferred Reporting Items for Systematic Reviews and Meta-Analyses (PRISMA) guidelines for reporting systematic reviews will be applied [19]. A flow diagram will be used to illustrate the literature search and article selection process, and a table will be compiled to provide an overview of the articles included in the review along with their characteristics. Furthermore, a map of sub-Saharan Africa with colors to show disease prevalence in each country will be included. For quantitative data, where possible, odds ratios (for categorical outcome data) or standardized mean differences (for continuous data) and their 95% CIs will be calculated from the data generated by each included study.

## Results

The outcome measures will include the prevalence of DCM and cardiovascular risk factors associated with DCM among patients with heart failure in sub-Saharan Africa. The anticipated or actual start date is January 1, 2021 and the anticipated completion date is April 30, 2021.

## Discussion

As CVDs are on the rise in sub-Saharan Africa [1,4,15], there is an urgent need to obtain more insight into the characteristics of the underlying pathologies in the region. It is currently unclear how heterogeneous or homogenous data on prevalence and risk factors for DCM are in sub-Saharan Africa, since previous reviews from the region addressed the prevalence and etiology of heart failure [2,6] or cardiomyopathies in general [9,16]. Likewise, it is unclear what paucity of data do exist regarding DCM in the region. To the best of our knowledge, no previous review is available with exclusive focus on DCM as an entity of heart failure in sub-Saharan Africa. We will discuss the literature on DCM in sub-Saharan Africa, describing the current status of DCM based on prevalence and cardiovascular risk factors, thus identifying gaps that need to be addressed to improve our understanding of DCM, with the overall goal to improve the prevention and management of this condition.

## Appendix

Multimedia Appendix 1 ROBINS-I tool.

Multimedia Appendix 2 Data extraction tool adapted from the Cochrane Collaboration tool.

## Abbreviations

CVD: cardiovascular disease
DCM: dilated cardiomyopathy
ESC: European Society of Cardiology
PRISMA: Preferred Reporting Items for Systematic Reviews and Meta-Analyses
