# Supplementary material for: Risk Factors and Prevalence of Dilated Cardiomyopathy in Sub-Saharan Africa: Protocol for a Systematic Review
Source: JMIR Res Protoc. 2021 Jan 21;10(1):e18229. doi: 10.2196/18229 (PMC7862000; doi:10.2196/18229)
Supplement: Multimedia Appendix 2 [file resprot_v10i1e18229_app2.pdf]

Data Extraction Form adapted from the Cochrane Collaboration

Title of the systematic review: Risk Factors and Prevalence of Dilated Cardiomyopathy in Sub-Saharan Africa: Protocol for a Systematic Review

Notes on using this data extraction form:

- Be consistent in the order and style you use to describe the information for each included study
- Record any missing information as unclear or not described, to make it clear that the information was not found in the study report(s), not that you forgot to extract it.
- Include any instructions and decision rules on the Data Extraction Form, or in an accompanying document. It is important to practice using the form and give training to any other authors using the form.
- We will protect the document in order to use the form fields (Tools / Protect document)

1. General Information

1. **Date form completed** *(dd/mm/yyyy)*	
2. **Name/ID of person extracting data**	
3. **Report title** *(title of paper/ abstract/ report that data are extracted from)*	
4. **Report contact details of person extracting data**	
5. **Publication type** *(e.g. full report, abstract, letter)*	
6. **Study ID** *(e.g. 01 plus surname of first author and year first full report of study was published e.g. Smith 2001)*	
7. **Country in which the study conducted**	

9. **Study funding source** *(including role of funders)*	
10. **Possible conflicts of interest** *(for study authors e.g. not reported)*	
11. **Notes:**	

2. Eligibility

**Study Characteristics**	**Review Inclusion Criteria** *(Insert inclusion criteria for each characteristic as defined in the Protocol e.g. cross-sectional, cohort or case-control)*	**Location in text** *(page#/fig /table)*
12. **Type of study**		
13. **Population description**		
14. **Focused diseases / conditions**		
15. **Types of outcome measures** *(Prevalence/Risk factors)*		
16. **Decision** *(with reasons for either inclusion or exclusion)*		
17. **Notes:**		

DO NOT PROCEED IF STUDY IS EXCLUDED FROM REVIEW

3. Population and setting

	**Description**	**Location in text** *(page#/fig /table)*
18. **Population description** *(from which study participants are drawn)*		

	Description	Location in text *(page#/fig /table)*
19. **Source/setting of the population** *(e.g. urban, rural, particular ethnic group)*		
20. **Method/s of recruitment of participants**		
21. **Notes:**		

4. Methods

	Descriptions as stated in report/paper	Location in text *(page#/fig /table)*
22. **Aim of study**		
23. **Design** *(e.g. cross-sectional study, cohort study, case-control study)*		
24. **Sampling technique** *(e.g. random or convenience)*		
25. **Study start date**		
26. **Study End date/duration** *(if any cohort)*		
27. **Notes:**		

5. Participants

	Description as stated in report/paper	Location in text *(page#/fig /table)*
28. **Total number of participants/Sample size**		
29. **Age group**		

6. Outcomes

**How outcomes measured**	Description as stated in report/paper	Location in text *(page#/fig/ table)*
30. **Outcomes**		

Copy and paste table for each outcome.

**Outcome 1: Prevalence**<br><br>**(Note:** Not detail here under outcome. Detail should be reported in results section**)**	**Description as stated in report/paper**	**Location in text** *(page#/fig /table)*
31. **Outcome names**		
32. **Time points measured** *(report the start year/specify whether from start and end of intervention)*		
33. **Time points reported**		

**Outcome 1: Prevalence**<br><br>**(Note:** Not detail here under outcome. Detail should be reported in results section**)**	**Description as stated in report/paper**	**Location in text** *(page#/fig /table)*
34. **Outcome definition**		
35. **Type of measurement** *(Percentage/Odds ratio/Risk ratio)*		
36. **Is outcome/tool validated?** *(Yes/No/Unclear/Not mentioned)*		
37. **Notes:**		

**Outcome 2:** Risk factors<br><br>(not detail here)	**Description as stated in report/paper**	**Location in text** *((page#/fig /table)*
38. **Name of the risk factors** ***Age, gender, family history, hypertension, diabetes, tobacco use, physical inactivity, poor nutrition, excessive alcohol consumption, high cholesterol and obesity***		
39. **Time points measured** ***(report the start year/specify whether from start and end of intervention)***		
40. **Type of measurement** *(Percentage/Odds ratio/Risk ratio)*		
41. **Is outcome/tool validated?** *(Yes/No/Unclear/Not mentioned)*		
42. **Notes:**		

7. Results and findings

Copy and paste the appropriate table for each outcome, including additional tables for each time point and subgroup as required.

**Outcome 1: Prevalence**<br><br>**(Note:** detail here**)**	**Description as stated in report/paper**	**Location in text** *(page#/fig /table)*
43. **Outcome**		
44. **Subgroup** *(if any, e.g. age-specific prevalence reporting)*		
45. **Results**		
46. **Response/non-response rate**		
47. **Any other results reported**		
48. **Unit of analysis** *(e.g. by individuals)*		
49. **Statistical methods used and appropriateness of these methods** *(e.g. proportion/%s, RR/OR)*		
50. **Whether results weighted?** *(e.g. Yes/No)*		
51. **Notes:**		

**Outcome 2: Risk factors**<br><br>**(Note:** detail here**)**	**Description as stated in report/paper**	**Location in text** *(page#/fig /table)*
52. **Name of the risk factors**		
53. **Results**		
54. **Response/non-response rate**		
55. **Any other results reported**		
56. **Unit of analysis** *(e.g. by individuals)*		

**Outcome 2: Risk factors**<br><br>**(Note:** detail here**)**	**Description as stated in report/paper**	**Location in text** *(page#/fig /table)*
57. **Statistical methods used and appropriateness of these methods** *(e.g. proportion/%s, RR/OR)*		
58. **All systematic and random error adjusted?** *(e.g. confounding, effect medication etc.)*		
59. **Notes:**		

8. Limitation and mitigation strategy

	Description as stated in report/paper	Location in text*(page# /fig/table)*
60. **Strength**		
61. **Limitation**		
62. **Strategies to overcome the limitation**		
63. **Notes:**		

9. Conclusion and other information

	**Description as stated in report/paper**	**Location in text** *(page#/fig /table)*
64. **Key conclusions of study authors**		
65. **Notes:**		
